# The Evaluation of Professional Divisions of Traditional Chinese Medicine in Taiwan through Patient Visit Records of 2012

**DOI:** 10.3390/ijerph15091992

**Published:** 2018-09-13

**Authors:** Ta-Peng Wu, Cheng-Hung Tsai, Yu-Ting Su, Chu-Chiao Wang, Tzeng-Ji Chen, Ching-Mao Chang, Fang-Pey Chen

**Affiliations:** 1 Center for Traditional Chinese Medicine, Taipei Veterans General Hospital, No. 201, Section 2, Shipai Road, Beitou District, Taipei City 11217, Taiwan; tpwu@vghtpe.gov.tw (T.-P.W.); voeitsai@gmail.com (C.-H.T.); u100023302@cmu.edu.tw (Y.-T.S.); wang801117@gmail.com (C.-C.W.); magicbjp@gmail.com (C.-M.C.); 2 School of Chinese Medicine, China Medical University, No.91, Hsueh-Shih Road, North District, Taichung 40402, Taiwan; 3 Faculty of Medicine, National Yang-Ming University, No.155, Section 2, Li-Nong Street, Beitou District, Taipei City 11221, Taiwan; tjchen@vghtpe.gov.tw; 4 Department of Family Medicine, Taipei Veterans General Hospital, Taipei City 11217, Taiwan; 5 Institute of Traditional Medicine, School of Medicine, National Yang-Ming University, No.155, Section 2, Li-Nong Street, Beitou District, Taipei City 11221, Taiwan

**Keywords:** traditional Chinese medicine, National Health Insurance, professional divisions

## Abstract

For decades, professional divisions have been represented as the main structural divisions in Western medicine throughout the world. In Taiwan, medical policymakers are also interested in designing professional divisions of traditional Chinese medicine (TCM). Therefore, this study evaluated the current status and potentiality of professional divisions of TCM in Taiwan using data from the year 2012 obtained from the National Health Insurance Research Database; the database provides information regarding age and gender of TCM physicians (TCMPs); total visit counts; contracted medical institution codes; groupings of diseases classified under International Classification of Diseases, Ninth Revision, Clinical Modification codes; numbers of children and female patients seeking treatment; and claim disposition codes used by each TCMP. The results indicated that there were 5522 TCMPs in 2012, and 4876 (90.3%) TCMPs practiced in primary clinics. The proportions of pediatric visits to these TCMPs were mostly below 0.2, and acupuncture or traumatology-related visit proportions were below 0.5. Only a few of the studied Taiwan-based TCMPs practiced gynecology and pediatrics, but most of them performed “internal medicine”, or “acupuncture” or “traumatology” treatments. Thus, the number of TCM specialists practicing gynecology or pediatrics is insufficient, indicating that a policy that forms professional divisions of TCM practitioners in Taiwan should be reconsidered.

## 1. Introduction

Although Western medicine has been classified into four main professional specialties and many subdivisions, the classification of traditional Chinese medicine (TCM) can be divided into only two categories: internal herbal medicine, and external procedures (acupuncture, moxibustion, manipulative therapy, etc.). The official classification of TCM was first introduced in 624 AD. The Imperial Medical Bureau of Tang dynasty classified TCM into four main subjects: “medicine”, “acupuncture”, “massage (which encompasses orthopedics)”, and “charms and incantations”, and practitioners were classified into five categories, with “pharmacy” as the fifth category. Medicine was the largest division and was further subdivided into five departments: “physical therapy”, “sores therapy”, “pediatrics”, “ear, eye, mouth and teeth” and “cupping therapy” [1]. During the Song dynasty, pediatric medicine entered a new era with the publication of *Key to Therapeutics of Children’s Diseases* by Yi Qian [2]. By 1076 AD, the Imperial Medical Bureau of Song dynasty had evolved into an educational institution for medicine and was divided into nine subjects: “internal medicine”, “pediatrics”, “wind-related”, “ophthalmology”, “gynecology”, “acupuncture and moxibustion”, “sores and ulcers”, “throat and teeth”, and “war injuries and spells” [3,4], indicating that TCM had more specific professional divisions then compared with modern times.

During Song dynasty, the increased application of Chinese remedies caused TCM to lean more toward internal medicine [5]. At this time, surgical procedures in TCM were limited because they were mostly based on the anatomical observation of muscles. In addition, the “spleen governing the muscles” concept was proposed by Gao Li, one of the four eminent physicians during the Jing and Yuan period in China. His book “Treatise on the Spleen and Stomach” led to “spleen governing the muscles” becoming the leading concept in the medical field during Yuan, Ming and Qing dynasties in China and even became the core of surgery-related professional divisions. Pulse detection and Chinese remedies have replaced most surgical procedures in TCM for treatment since then [6]. This subsequently led to difficulty in establishing a system that used surgery as the therapeutic method: surgery was also perceived as being technical, skill-dependent, or a form of superstition. Acupuncture, moxibustion, ophthalmologic treatment, and other surgical opening and stitching techniques were deemed to be fading trends like witchcraft, and were gradually marginalized or excluded [7]. During Ming and Qing dynasties, the wide use of herbal remedies further blurred the distinctions between professional divisions of TCM because TCM was largely influenced by the belief that the functions of all internal organs in the human body are interdependent, and a holistic balance must be achieved in the therapeutic approach [6]. In addition, TCM physicians (TCMPs) did not require any qualifications or licenses, with the exception of imperial physicians in the palace during Ming and Qing dynasties [8].

In the early period of the Republic of China (ROC), although most expert TCMPs were trained through master–apprentice education [9], medical treatments were carried out by medical quacks [10], witch practitioners [11], and through the drawing of divination lots in temples [12,13] in the countryside of China. In Taiwan, issuing certificates to qualified TCMPs was discontinued in 1902 AD, causing a rapid decrease in the number of TCMPs during the period of Japanese governance, but the use of Chinese remedies did not decline [14]. In 1966, after the ROC government retreated, the Department of traditional Chinese medicine was established at the China Medical College (now known as China Medical University), Taiwan, and in 1968, the first qualification examination for TCMPs was held [15]. The TCM education course was 7 years, and students were expected to pass the national certification examination before they could obtain a license to practice. According to the early policy of the Ministry of Health (now the Ministry of Health and Welfare) in Taiwan, professional divisions of TCM consist of internal “medicine”, “dermatology”, “pediatrics”, “gynecology”, “traumatology”, “acupuncture and moxibustion”, “hemorrhoids”, and “ophthalmology”. Since National Health Insurance (NHI) was established in 1996, under the payout system, all professional divisions are classified as simply TCM, and the claim codes indicate that “internal medicine”, “acupuncture”, and “traumatology” are the only subdivisions of TCM in Taiwan [16].

The Executive Yuan of Taiwan implemented the NHI on 1 March 1995, and initially covered Western medicine and dentistry. TCM was subsequently covered in 1996, and treatment by only accredited and licensed TCMPs in either outpatient departments or primary clinics was claimable under the NHI system [17]. Since then, reimbursement for TCM outpatient services, such as granule prescriptions (made from herbs by a pharmaceutical company with good manufacturing practices), acupuncture, moxibustion, and manipulation therapy (belongs to the scope of traumatology in TCM), is fully covered by NHI. Under the NHI system, the insurance coverage rate is approximately 99% of the population, but copayment and registration fees must be paid by patients [18]. The NHI does not cover TCM services, such as prescription for raw herbs, cupping, Gua sha, and bloodletting, and does not cover inpatient stays. The NHI Research Database (NHIRD) has been operational for 22 years. It contains data from each year and is used for many research topics, such as clinical studies, biomedical sciences, and medical-economic sciences [19]. An increasing number of research papers that are pertinent to TCM have been published based on data from the NHIRD, and many have been included in influential journals.

Most TCMPs in Taiwan are general practitioners, and they provide TCM services at primary clinics in communities [20]. During the years 1996 to 2001 in Taiwan, 82.6% of the TCM services provided were at primary clinics, whereas only 12.8% were at private and government hospitals [21]. For health care policymakers, the differentiation of the professional divisions of TCM is required at medical centers and hospitals. Since TCM has been popular in Taiwan, and there are TCMPs with license and college education leading it to move closer to Western medicine. However, many subspecialties exist in Western medicine, but TCMPs have not been able to establish professional divisions. Under such circumstances, comprehensively analyzing the current status of the professional certification of TCMPs in Taiwan is crucial for establishing a professional division system of TCM practitioners, and NHIRD data from the year 2012 were used in this study to achieve this goal.

## 2. Study Design

### 2.1. Database

Taiwan’s NHI is government-administered and compulsory for all citizens. In 2012, the NHI covered approximately 23 million people, 99.89% of the total population [22].

In this study, NHIRD data from the year 2012 data were analyzed; these were related to practicing TCMPs and contained information on their age, sex, services provided, total number of outpatient services provided in that year, and the International Classification of Diseases, Ninth Revision, Clinical Modification (ICD-9-CM) codes of outpatient services they provided. These data were then analyzed using statistical software to identify trends regarding treatments provided and determine whether professional divisions were evident. The NHI administration made the NHIRD claims data publicly available for researchers from 1996 to 2013. This study was approved by the Institutional Review Board of Taipei Veterans General Hospital (2013-10-001CE).

### 2.2. Research Population

A descriptive and cross-sectional analysis on the 2012 data was conducted. Of the 5524 certified TCMPs in Taiwan, two records were incomplete and excluded, resulting in 5522 TCMPs being studied. The following data were analyzed: the age and sex of each TCMP, total visit count each TCMP registered with the NHI, contracted medical institution codes, the ICD-9-CM codes used by each TCMP for health insurance claims, number of children seeking treatment, number of female patients seeking treatment, and the claim codes used by each TCMP. The analysis determined the ratios of children, female patients, patients seeking acupuncture treatment, patients seeking traumatology treatment, and patients with various diseases (as classified under the ICD-9-CM codes) seeking TCM treatment.

Contracted medical care institutions in this study, as defined by the NHI, comprised four types: “academic medical centers”, “metropolitan hospitals”, “local community hospitals”, and “physician clinics (known as primary clinics)”.

### 2.3. Data Processing

Data were processed using Perl software (version 5.20.2) and descriptive statistics was generated using Microsoft Excel 2013. The results are presented in the figures provided in this paper. We expressed the proportions of specific patient types, diagnoses, or treatment methods (e.g., acupuncture) of a particular TCMP as a ratio labeled as a proportion (e.g., acupuncture proportion) of the total visit counts, and we used this to determine if that TCMP could be characterized according to visitation trends. For example, by dividing the number of reported pediatric visits by the total visit count, we obtained the proportion of pediatric visits (pediatric visits/total visits = pediatric visit proportion) of a particular TCMP. A high pediatric visitation ratio indicates that a considerable proportion of a particular TCMP’s patients were children. The same principle was applied to female patients. However, not all female patients visited physicians for gynecological treatments. Hence, female patients with ICD-9-CM codes corresponding to “diseases of female genital organs” (614–629) were further segregated and expressed as a ratio of total visit counts to obtain the proportion of women who went for gynecological treatments. A similar segmentation approach was used for patients with claim codes corresponding to acupuncture or traumatology treatment. Finally, the same method was used to determine and analyze the visitation ratios for various groupings of diseases classified under ICD-9-CM codes (see Table 1 for the systemization of ICD-9-CM codes).

## 3. Results

**Figure 1:**
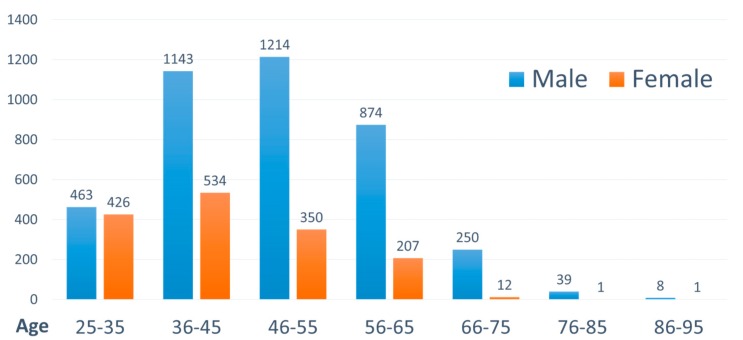
Age and sex distribution of traditional Chinese medicine physicians (TCMPs) in Taiwan in 2012.

A total of 5522 practicing TCMPs in 2012 were selected for the analysis. Of them, 1531 were women. The studied TCMPs were aged between 25 and 95 years (Figure 1). As displayed in the figure, for those between 35 and 75 years old, the ratio of male to female physicians was large, at 3.51:1 (3481:1003); however, for those between 25 and 35 years old, the ratio was closer to 1:1 (463:426), indicating an increase in the number of young female TCMPs.

**Figure 2:**
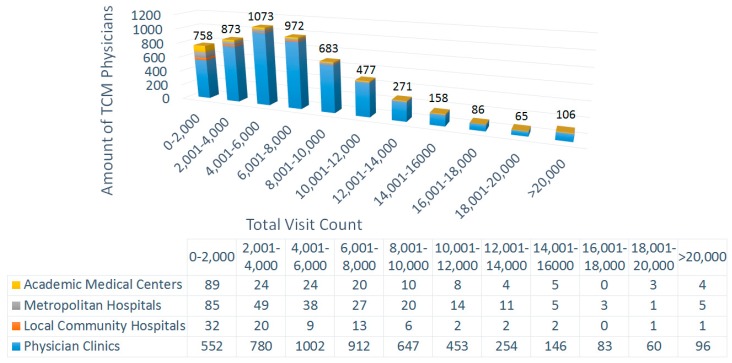
Analysis of total traditional Chinese medicine (TCM)-related visit counts and medical institution type in which care was provided by each TCMP in Taiwan in 2012.

As shown in the distribution chart (Figure 2), 4985 TCMPs (90.3%) provided services in primary clinics. Most of the TCMPs (78.9%) had a total visit count lower than 10,000, and 87.5% of TCMPs had a total visit count lower than 12,000; and the peak range for total visit count was 4001–6000 visits. The TCMPs that mostly provided services in academic medical centers had visit counts below 2000 (89 physicians, 46.6%). For the 106 TCMPs with total visit counts above 20,000, 90.6% worked in primary clinics.

**Figure 3:**
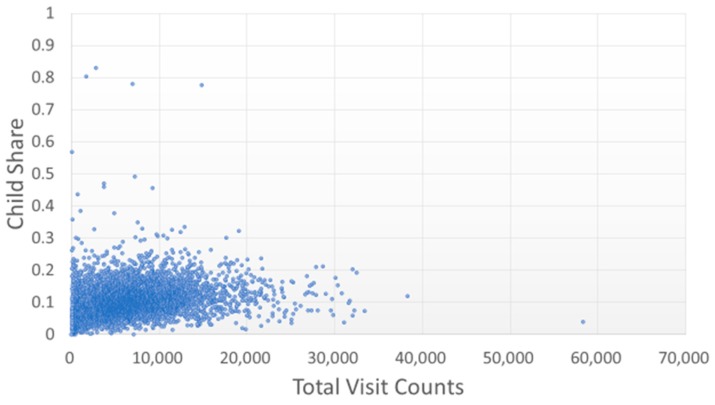
Distribution of pediatric visits with total visit counts by each TCMP. A higher pediatric visitation ratio indicates that the TCMP treated more children.

**Figure 4:**
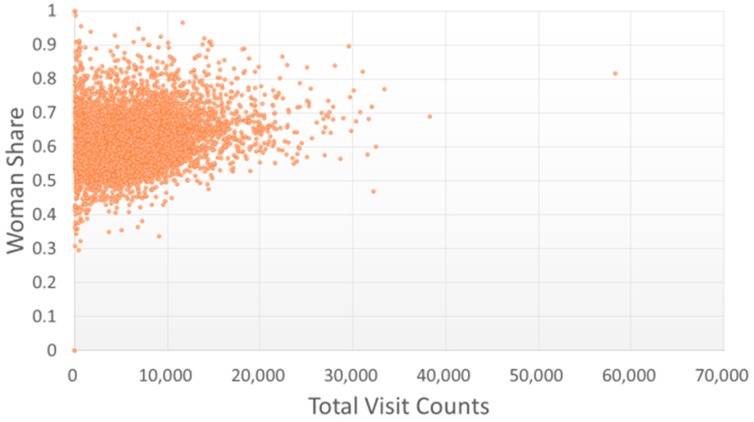
Distribution of female patient visits with total visit counts by each TCMP. A higher ratio of female patients indicates that the TCMP treated more female patients.

**Figure 5:**
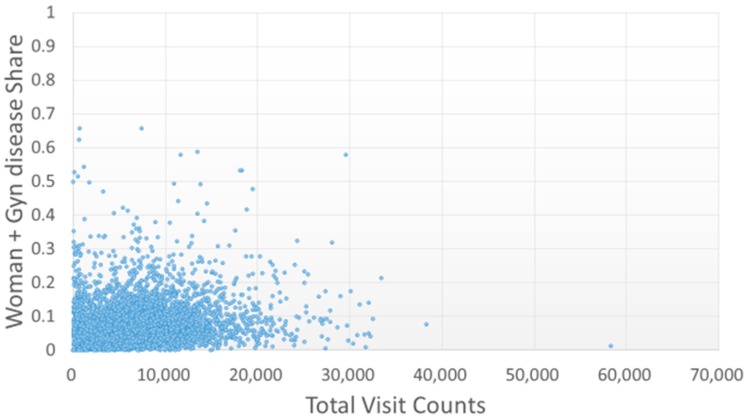
Distribution of gynecology-related treatments with total visit counts by each TCMP. A higher proportion indicates that the TCMP provided more gynecological treatments to female patients (Gyn disease).

Most TCMPs had a pediatric visitation ratio lower than 0.2 (Figure 3), and only five TCMPs had a pediatric visitation ratio above 0.5, indicating that one in every two patients that visited the TCMPs was a child. Most TCMPs had a female patient ratio of 0.4–0.8 (Figure 4). However, the ratio of gynecology-related visitations of TCMPs was lower than 0.2 (Figure 5), indicating that most female patients did not visit TCMPs for gynecological treatments.

**Figure 6:**
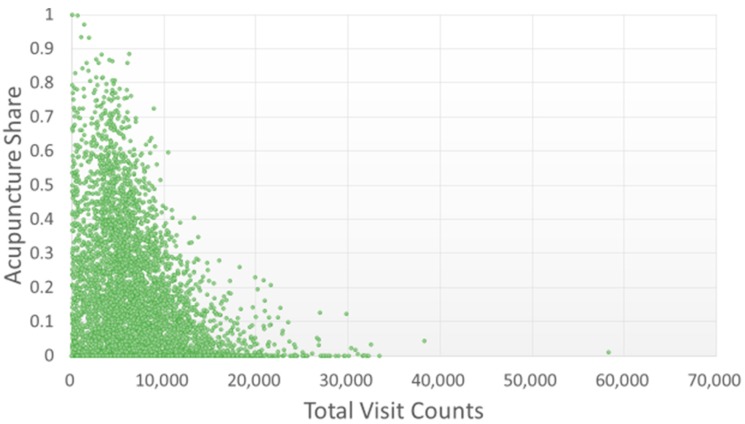
Distribution of acupuncture treatments with total visit counts by each TCMP. A larger acupuncture proportion indicates that the TCMP administered more acupuncture treatments to patients.

**Figure 7:**
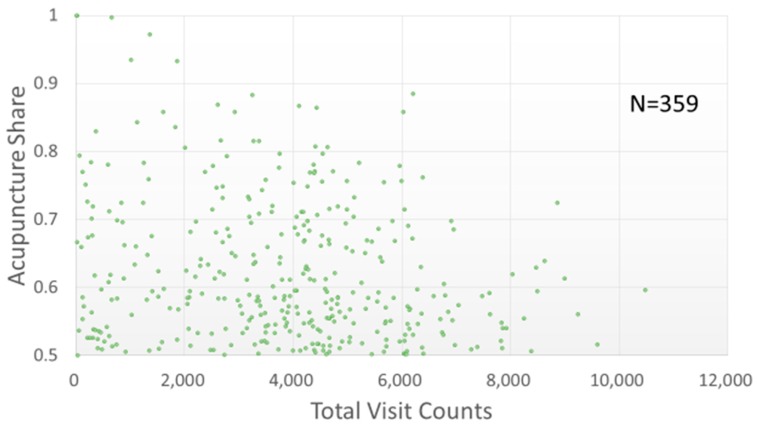
Distribution of acupuncture proportion (above 0.5) with total visit counts by each TCMP. The TCMPs represented in this figure administered acupuncture therapy to more than half of their patients.

**Figure 8:**
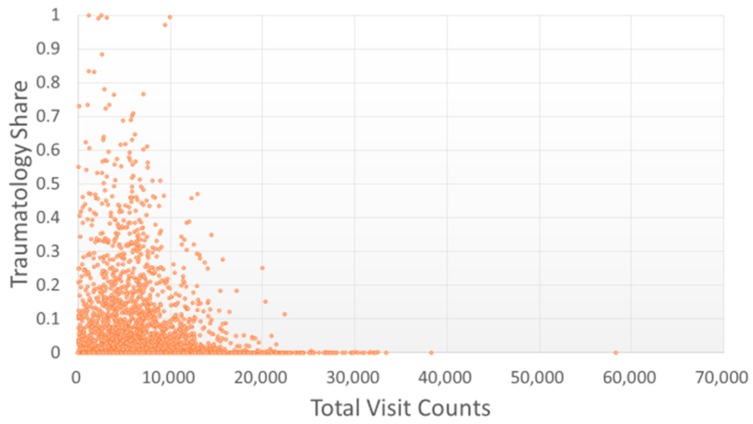
Distribution of traumatology treatments with total visit counts by each TCMP. A larger proportion indicates TCMPs using more traumatology-related manipulative therapy on patients.

**Figure 9:**
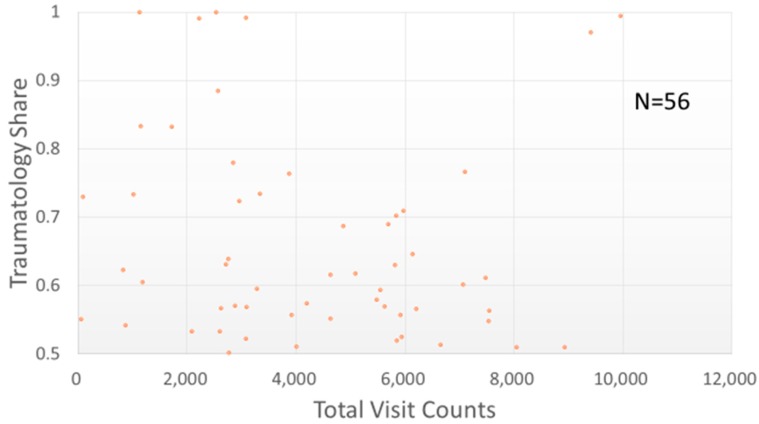
Distribution of traumatology proportion (over 0.5) with total visit counts by each TCMP. The TCMPs represented in this figure used traumatology-related manipulative therapy to treat more than half of their patients.

**Figure 10:**
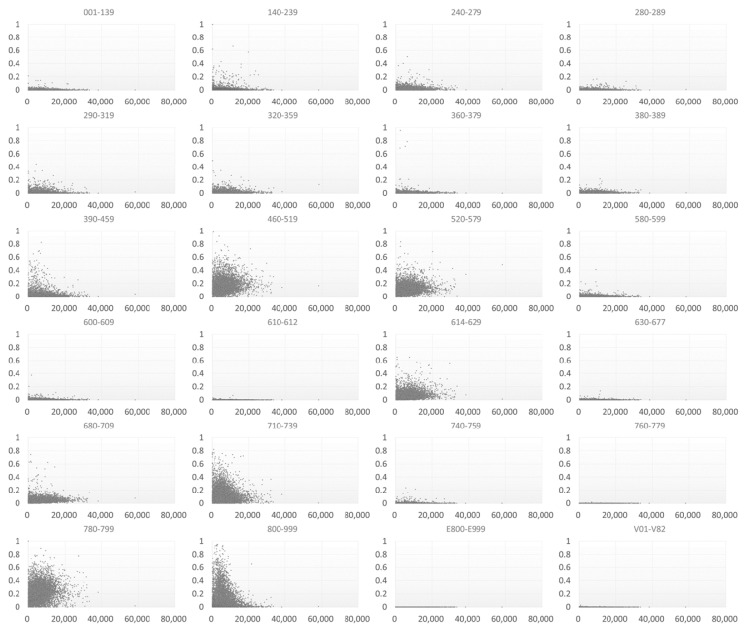
Distribution of ICD-9-CM codes with total visit counts by each TCMP. A larger proportion indicates that TCMPs used more ICD-9-CM codes within a specific range in patient data.

**Figure 11:**
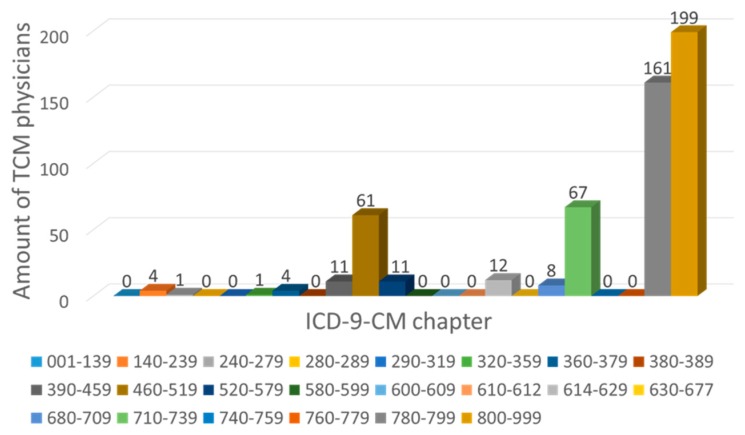
Total number of TCMPs with every ICD-9-CM grouping proportion over 0.5 only. The TCMPs in this figure used a specific ICD-9-CM code to treat more than half of the patients.

Most of the studied TCMPs had acupuncture treatment ratios smaller than 0.5 (Figure 6). Of the 359 physicians with acupuncture treatment ratios larger than 0.5 (i.e., more than one in every two patients visited the physician for acupuncture), only one TCMP had a total visit count of over 10,000 (Figure 7). The traumatology treatment proportion is shown in Figure 8. Of the 56 TCMPs with traumatology ratios above 0.5, none had a total visit count exceeding 10,000 (Figure 9). Additionally, as depicted in Figure 10, most of the ICD-9-CM codes for the TCM patients were categorized under “Diseases of the Respiratory System”, “Diseases of the Digestive System”, “Diseases of the Skin and Subcutaneous Tissue”, “Diseases of the Musculoskeletal System and Connective Tissue”, or “Diseases of the Female Genital Organs” groupings. Proportions of patients with ICD-9-CM codes falling under “Symptoms, Signs, and Ill-Defined Conditions”, and the “Injury and Poisoning” grouping were also high. “Infectious and Parasitic Diseases”, “Neoplasms”, “Endocrine, Nutritional and Metabolic Diseases and Immunity Disorders”, “Diseases of the Blood and Blood-Forming Organs”, “Mental Disorders”, “Diseases of the Nervous System”, “Diseases of the Eye and Adnexa”, “Diseases of the Ear and Mastoid Process”, “Diseases of the Circulatory System”, “Diseases of the Urinary System”, “Diseases of the Male Genital Organs”, “Disorders of Breast”, “Complications of Pregnancy, Child Birth, and the Puerperium” and “Diseases of the Skin and Subcutaneous Tissue” groupings were comparatively few. Of the TCMPs with grouping proportions higher than 0.5 (Figure 11), 199 TCMPs applied the most ICD-9-CM codes from “Injury and Poisoning”, 161 from “Symptoms, Signs and Ill-Defined Conditions”, 67 from “Diseases of the Musculoskeletal System and Connective Tissue”, 61 from “Diseases of the Respiratory System”, 12 from “Diseases of Female Genital Organs”, 11 from “Diseases of the Circulatory System” and “Diseases of the Digestive System”, 8 from “Diseases of the Skin and Subcutaneous Tissue”, 4 from “Neoplasms” and “Diseases of the Eye and Adnexa” and 1 from “Endocrine, Nutritional and Metabolic Diseases and Immunity Disorders” and “Diseases of the Nervous System”. No other grouping proportions calculated from TCMP-applied ICD-9-CM codes were higher than 0.5.

## 4. Discussion

Research findings obtained in 2007 indicate that the TCMP resources in Taiwan were saturated with the natural growth of their supply, and the average number of visits per TCMP decreased annually [23]. In 2003, scholars predicted that the number of TCMPs in 2010 would be 4606 [24]. According to our study, there were 5522 licensed TCMPs in 2012, and 758 (13.7%) exhibited a total visit count of under 2000 per year (Figure 2). Based on the NHI payout records, every patient paid equal fees per visit regardless of consultation and treatment time, indicating that only a higher visit count would increase payout amount. Determining whether the aforementioned physicians with low visit counts had treated self-paying patients to increase their income was difficult because data from the NHI database were used for this study.

Because of the increased education level of women and promotion of gender equality, an increase in the proportion of female TCMPs among those aged under 35 years was observed, with the ratio of male to female physicians approaching 1:1. However, the proportion of male TCMPs was higher for older age groups. More female patients sought TCM treatments than male patients [25,26,27]. Thus, visiting female TCMPs might decrease their anxiety during treatment and avoid related medical conflicts.

According to the aforementioned regulations in Taiwan, various professional divisions of TCM could be registered. However, in this study, from the analysis of claim codes or the categorization of patients into children, women, and women with gynecology-related diagnostic codes, few TCMPs focused on pediatrics or gynecology (Figure 3 and Figure 5). Therefore, the number of qualified teachers promoting TCM gynecology and pediatrics might be insufficient.

Currently, TCM in Taiwan is categorized into three main subspecialties: “internal medicine” (involving pediatrics and gynecology), “acupuncture”, and “traumatology”. As illustrated in Figure 6 and Figure 7, the TCMPs relevant to acupuncture and traumatology treatments exhibited relatively low total patient visit counts. This might be the reason why these physicians spent longer treating patients. Huang reported that only 8.9% of TCM students would like to practice traumatology after graduation [28] because of the long working hours, difficulty of techniques, high medical risks, and low income levels [29]. If future policies were to promote professional divisions of TCM, attention should be given to acupuncture and traumatology because they may encounter difficulties in recruitment and cause students to change their specialties before completing their training, leading to scarcity of physicians in these fields. In other words, income and cost of risk influence a doctor’s choice of professional divisions. Although professional divisions of TCM enable patients to choose a physician who is experienced in the specialty, TCM focuses on holistic concept of a human being, and a patient may express multiple complaints regarding many organs of the body that may not be limited to a single professional division. In these cases, professional divisions of TCM may cause complications during treatment and wastage of medical resources.

Professional divisions of TCM are more feasible in hospitals (academic medical centers, metropolitan hospitals, and local community hospitals) than in primary clinics because critically ill patients will not visit primary clinics. However, 80% of TCM patients visited primary clinics [21], and 90.3% of the TCMPs practiced in such clinics (Figure 2). In addition, few patients sought TCM treatments in hospitals. Furthermore, TCMPs play a similar role as family doctors of Western medicine, and they are typically at the frontline of general public health care. Therefore, we observed that TCMPs used the ICD-9-CM code grouping “Symptoms, Signs and Ill-Defined Conditions” to a great extent (Figure 10 and Figure 11). Furthermore, diagnoses from TCMPs are also typically unclear, hindering professional divisions of TCM.

Studies have revealed that the most commonly used ICD-9-CM codes for NHI TCM claims were related to the “Diseases of the Respiratory System” [21,30,31]. Most patients who “shop” towards TCMPs seek treatment for respiratory diseases (44.7%), followed by sprains and injuries (44.0%) [32], and these patients also attended otolaryngology clinics (known as ENT clinics) and Western medicine physical therapy clinics [32]. Additionally, published results have detailed information regarding the most commonly used ICD-9-CM codes and prescriptions [33].

Some limitations exist in this study, which are recapitulated in the following points. Firstly, this study used single-year data from the NHIRD, which did not involve self-paid treatment procedures or decoctions. The information extracted from the NHIRD did not represent all TCM treatments in Taiwan. In addition, Taiwanese people can purchase Chinese herbs and remedies from pharmacies without visiting any TCMP; therefore, self-paid Chinese remedies were not been recorded in the NHIRD [31]. Research indicates that more than half of the patients seeking TCM treatment were willing to self-pay for only a few types of services and preferred to claim health insurance for most services, and only one-fourth of the patients interviewed opted for copayment. This was because patients could not judge the quality of medical products, were unsure whether the quality matched the price paid, were worried about the financial burden, or were worried that they might have purchased counterfeit drugs [34]. Therefore, a high proportion of self-pay procedures was deemed as unlikely. Secondly, the NHIRD has several limitations. For example, a short consultation time under the health insurance system results in insufficient recorded details [18]. Thirdly, in addition, similar to research based on observations, results that are not obtained experimentally cannot provide conclusive evidence for causation. However, research data obtained from the analysis of large databases could still be of value to clinical studies because the database contains information from a nationwide population [35], which overcomes the difficulty of obtaining statistically significant results.

Because the NHI covers most diseases and treatments, this study demonstrated the distinct characteristics of TCM outpatients, which are crucial in the medical field. These data can serve as references for future policymakers. This study was also free from selection or recall biases because of the structure of the samples and database. Due to the large sample size, this study achieved high statistical power and precision, and therefore provided a basis for conducting further international studies of medical policies.

## 5. Conclusions

This novel study investigated the trends of professional divisions among TCMPs in Taiwan. The 2012 NHIRD data were adopted to analyze the claim codes and ICD-9-CM codes of the patients in addition to other factors for each TCMP, thereby determining whether a tendency existed toward the development of professional divisions of TCMPs. The results implied that TCM in Taiwan is currently categorized in three main specialties: “internal medicine”, “acupuncture”, and “traumatology.” The number of TCMPs specializing in pediatrics and gynecology, which are subspecialties in internal medicine, remains limited, indicating that detailed professional divisions in the future may lack sufficient physicians to treat related patients. These findings provide a reference for TCM for health authorities so that they can plan regulations and policies concerning TCM and satisfy the training requirements of clinical professional divisions.

## Figures and Tables

**Table 1:** Grouping system of ICD-9-CM codes.

Range of ICD-9-CM Codes	Description for Each Group
001–139	Infectious and Parasitic Diseases
140–239	Neoplasms
240–279	Endocrine, Nutritional, and Metabolic Diseases and Immunity Disorders
280–289	Diseases of the Blood and Blood-forming Organs
290–319	Mental Disorders
320–359	Disorders of the Nervous System
360–379	Disorders of the Eye And Adnexa
380–389	Diseases of the Ear And Mastoid Process
390–459	Diseases of the Circulatory System
460–519	Diseases of the Respitory System
520–579	Diseases of the Digestive System
580–599	Diseases of Urinary System
600–609	Diseases of Male Genital Organs
610–612	Disorders of Breast
614–629	Diseases of Female Genital Organs
630–677	Complications of Pregnancy, Child Birth, and the Puerperium
680–709	Diseases of the Skin and Subcutaneous Tissue
710–739	Diseases of the Musculoskeletal System and Connective Tissue
740–759	Congenital Abnormalities
760–779	Certain Conditions Originating in the Perinatal Period
780–799	Symptoms, Signs and Ill-defined Conditions
800–999	Injury and Poisoning
V01–V82	Supplementary Classification of External Causes of Injury and Poisoning
E800–E999	Supplementary Classification of Factors Influencing Health Status and Contact with Health Services

Note: ICD-9-CM = International Classification of Diseases, Ninth Revision, Clinical Modification.

## Data Availability

The following information was supplied regarding the deposition of related data: Raw data for this work was obtained by application from the NIHRD, Taiwan (http://nhird.nhri.org.tw/en/index.htm) and may not be shared according to the database’s rules governing use. Access to the data used in this study may be obtained by citizens of the ROC who fulfill the requirements of conducting research projects.
